# Vaccine-Mediated Activation of Human TLR4 Is Affected by Modulation of Culture Conditions during Whole-Cell Pertussis Vaccine Preparation

**DOI:** 10.1371/journal.pone.0161428

**Published:** 2016-08-22

**Authors:** Marieke E. Hoonakker, Lisa M. Verhagen, Elder Pupo, Alex de Haan, Bernard Metz, Coenraad F. M. Hendriksen, Wanda G. H. Han, Arjen Sloots

**Affiliations:** 1 Institute for Translational Vaccinology (Intravacc), Bilthoven, The Netherlands; 2 Department of Animals in Science and Society, Faculty of Veterinary Medicine, Utrecht University, Utrecht, The Netherlands; 3 Centre for Immunology of Infectious Diseases and Vaccines, National Institute for Public Health and the Environment, Bilthoven, The Netherlands; Instituto Butantan, BRAZIL

## Abstract

The potency of whole-cell pertussis (wP) vaccines is still determined by an intracerebral mouse protection test. To allow development of suitable *in vitro* alternatives to this test, insight into relevant parameters to monitor the consistency of vaccine quality is essential. To this end, a panel of experimental wP vaccines of varying quality was prepared by sulfate-mediated suppression of the BvgASR master virulence regulatory system of *Bordetella pertussis* during cultivation. This system regulates the transcription of a range of virulence proteins, many of which are considered important for the induction of effective host immunity. The protein compositions and *in vivo* potencies of the vaccines were BvgASR dependent, with the vaccine containing the highest amount of virulence proteins having the highest *in vivo* potency. Here, the capacities of these vaccines to stimulate human Toll-like receptors (hTLR) 2 and 4 and the role these receptors play in wP vaccine-mediated activation of antigen-presenting cells *in vitro* were studied. Prolonged BvgASR suppression was associated with a decreased capacity of vaccines to activate hTLR4. In contrast, no significant differences in hTLR2 activation were observed. Similarly, vaccine-induced activation of MonoMac-6 and monocyte-derived dendritic cells was strongest with the highest potency vaccine. Blocking of TLR2 and TLR4 showed that differences in antigen-presenting cell activation could be largely attributed to vaccine-dependent variation in hTLR4 signalling. Interestingly, this BvgASR-dependent decrease in hTLR4 activation coincided with a reduction in GlcN-modified lipopolysaccharides in these vaccines. Accordingly, expression of the *lgmA-C* genes, required for this glucosamine modification, was significantly reduced in bacteria exposed to sulfate. Together, these findings demonstrate that the BvgASR status of bacteria during wP vaccine preparation is critical for their hTLR4 activation capacity and suggest that including such parameters to assess consistency of newly produced vaccines could bring *in vitro* testing of vaccine quality a step closer.

## Introduction

*BordetelIa pertussis* is a Gram-negative pathogen that causes whooping cough in humans. As pertussis disease in children can be severe, development of whole-cell pertussis (wP) vaccines started soon after it was known how to cultivate the bacterium under laboratory conditions. The introduction of these vaccines on a large scale resulted in the control of epidemic pertussis disease [1, 2]. Although today wP vaccines have been replaced by acellular pertussis vaccines in most industrialized countries due to concerns regarding their reactogenicity, wP vaccines are still used in many countries in Latin America, Africa and Asia [1]. Furthermore, recent evidence points towards a higher efficacy of vaccination schemes including a first dose of wP compared to schedules solely using aP vaccines [3–6]. Along with lower costs of production [7], this will likely make these the pertussis vaccine of choice in many regions for the years to come. For lot release of wP vaccines, the use of the intracerebral challenge test, also known as the Kendrick test, is a regulatory requirement at this moment [8, 9]. As it is questionable whether this mouse model appropriately reflects human pertussis disease, the results using these animal tests are highly variable within and among laboratories [10] and there is concern with respect to animal welfare [11], novel *in vitro* alternatives to assess the quality of newly produced wP vaccine lots are urgently needed.

For the quality of wP vaccines, the bacterial cultivation process is considered crucial as growth conditions are known to affect gene expression in *B*. *pertussis* [12, 13]. Expression of most virulence genes, whose products are involved in pathogenesis, is controlled by a master regulatory system encoded by the *BvgASR* locus [14]. This regulatory system enables the bacterium to adapt to environmental changes. In response to conditions such as temperatures below 26°C or the presence of sulfate (MgSO_4_) or nicotinic acid, expression of most virulence genes is suppressed [15]. This state is referred to as the Bvg^-^ phase as opposed to the Bvg^+^ phase in which the majority of virulence proteins are expressed. Using mutants locked in either the Bvg^-^ or Bvg^+^ phase, it has been shown that bacteria in the Bvg^-^ phase are unable to survive *in vivo* and that the Bvg^+^ phase is required to cause respiratory infection in mice [16]. Importantly, the presence of many of the virulence proteins expressed in the Bvg^+^ phase in wP vaccines has also been associated with the induction of protective immune responses. It has been shown that the amount of virulence proteins in a vaccine based on outer membrane vesicles correlated with protection [17]. In another study, immunization with inactivated *B*. *pertussis* bacteria harvested during the logarithmic growth phase (considered to contain high amounts of virulence proteins) induced an immune response with higher protective capacity compared with inactivated bacteria harvested after logarithmic growth (considered to contain lower amounts of virulence proteins)[18]. In addition, other investigators were able to confirm that a decreased availability of nutrients in *B*. *pertussis* cultures after the logarithmic growth phase is associated with a lower expression of many virulence genes in a BvgASR-dependent manner [12, 13]. Taken together, these results strongly suggest that the composition (i.e. quality) and hence protective capacity (i.e. potency) of a wP vaccine can be influenced by the culture conditions used within the wP manufacturing process. By sensing differences in the culture conditions, the BvgASR system most likely plays an important role in controlling these vaccine characteristics. Although some of these reports directly link differences in wP vaccine composition to the potency of these batches in the intracerebral challenge model [18], not much is known about the immunological consequences of vaccination with wP vaccines produced from *B*. *pertussis* bacteria cultured under BvgASR-modulating conditions.

The potency of a vaccine depends on the type of adaptive immune response that is initiated and directed by antigen-presenting cells (APC) [19]. This requires proper activation of APC through recognition of conserved microbial structures by pathogen recognition receptors (PRR), including the Toll-like receptors (TLR). Although *B*. *pertussis* is known to produce ligands for both TLR2 and TLR4 [20, 21], TLR4 signalling in particular was found to affect the development of immune responses against *B*. *pertussis*, whereas TLR2 was not [22]. In addition, TLR4 was shown to be essential for protection against *B*. *pertussis* in mice [23]. The canonical ligand for TLR4 is lipopolysaccharide (LPS), a well-known component of wP vaccines. It has been shown that *B*. *pertussis* can substitute the phosphate groups of the lipid A moiety of its LPS with glucosamine (GlcN), a modification that leads to enhanced hTLR4 signalling and the secretion of pro-inflammatory cytokines [24]. The genes *lgmA*, *lgmB* and *lgmC* have recently been identified to encode the enzymes required for this GlcN modification of *B*. *pertussis* LPS [24–26]. Importantly, the expression of *lgmA* and *lgmB* was found to be regulated by BvgASR master regulatory system [27]. Since culture conditions (such as nutrient availability) can affect the BvgASR system, they might also affect LPS structure during cultivation of *B*. *pertussis* bacteria. As LPS is an important contributor to the wP vaccine-induced activation of APC, we hypothesised that culture condition-induced changes in Bvg phase could affect APC activation and thereby influence the induction of adaptive immune responses and vaccine potency.

In this study, we investigated the capacity of several *in vitro* methods to assess wP vaccine quality. To properly address the potential of these assays, we used a set of experimental wP vaccines of varying quality that were produced by deliberate addition of sulfate to the bioreactor cultures in order to modulate the BvgASR system. *B*. *pertussis* bacteria (vaccine strain 509) were harvested just before sulfate addition and at several time points afterwards, resulting in vaccine products that contain varying amounts of virulence proteins. Based on protein composition and *in vivo* potency testing (Metz *et al*., submitted for publication), the qualities of these wP vaccines were considered to range from good to poor (vaccine A_ref_—E). Previously, we showed that these vaccines differed in their capacity to induce activation of monocyte-derived dendritic cells (moDC) and MonoMac-6 (MM6) cells *in vitro* and demonstrated that these cellular platforms had considerable potential as *in vitro* alternatives to animal testing for the quality control of wP vaccines [28]. Here, we investigated the relative contribution of TLR2 and TLR4 to the activation of these cells and showed that prolonged cultivation in the presence of sulfate not only triggered alterations in the expression of known virulence proteins but also in the expression of genes associated with LPS modification, leading to variations in LPS structure. These modifications were associated with the ability of the wP vaccines to induce human TLR4 but not TLR2 signalling and therefore likely influenced activation of human APC. Taken together, these findings demonstrate the necessity to monitor vaccine quality after production, and more importantly, they provide a scientific basis to the use of cell-based assays to assess aspects of the immunological potency of wP vaccines *in vitro*.

## Materials and Methods

### Production of experimental wP vaccines of different quality

In this study, experimental wP vaccine batches were used that were based on cultivation of *B*. *pertussis* strain 509 (Intravacc, Bilthoven, The Netherlands). This clinical isolate was collected in 1963 and was used for the production of wP vaccine for the national vaccination program of the Netherlands until 2005. The wP vaccines were produced as described in detail elsewhere (Metz *et al*. submitted for publication). Briefly, all cultures were grown in chemically defined THIJS medium [29, 30] using a 3L bioreactor equipped with a Rushton stirrer (Applikon, Schiedam, The Netherlands) at a constant temperature of 35°C. After obtaining a steady-state culture, deliberate down-regulation of virulence genes (t = 0) was initiated by adding MgSO_4_ to the medium at final concentrations of 50 mM. Samples were taken just before the addition of MgSO_4_ (0 hours) and 2, 6, 12 and 24 hours after this addition, inactivated with formaldehyde (16 mM) and heating (56°C) for 10 min. The resulting wP vaccines batches are referred to as vaccine A_ref_ (t = 0) (reference vaccine), vaccine B (t = 2), vaccine C (t = 6), vaccine D (t = 12) and vaccine E (t = 24). Three separate cultivation runs were performed. Unless mentioned otherwise, corresponding vaccine preparations derived from the different runs were pooled and used for the stimulation of different cell lines or monocyte derived dendritic cells (moDC) at indicated OD_590nm_.

### Reagents

Ultrapure LPS from *E*. *coli* K12 (LPS-EC), ultrapure LPS from *Rhodobacter sphaeroides* (LPS-RS), PAM3CSK4 (PAM), HEK-Blue selection antibiotics, Normocin, Zeocin and QUANTI-Blue were all purchased from InvivoGen Europe (Toulouse, France). The α-TLR2 blocking antibody was obtained from R&D systems. Recombinant human GM-CSF was purchased from PeproTech (Rocky Hill, NJ, USA) and recombinant human IL-4 was purchased from Sanquin (Amsterdam, The Netherlands). The IL-6 ELISA kit was purchased from Sanquin, the IL-12p40 ELISA kit was purchased from Diaclone and the IL-8 ELISA kit was obtained from R&D systems. Dulbecco’s modified Eagle’s medium (DMEM) and Iscove’s modified Dulbecco’s medium (IMDM) were purchased from Gibco, FCS was obtained from Thermo scientific (Waltham, MA). Fatty acid standards C14:0, C14:0-3OH, NaOH, HCl and tert-butyl methyl ether were purchased from Sigma (Zwijndrecht, The Netherlands). Methanol and n-Hexane were from JT Baker and n-Hexane from Biosolve.

### ELISA for the specific detection of virulence antigens

In contrast to the ELISA that was performed directly after vaccine preparation (Metz *et al*., submitted for publication), here specific *B*. *pertussis* antigens (FHA, PRN, FIM2, FIM3, Vag8, PT, LPS) in vaccines Aref—E were quantified by ELISA approximately 1.5 years after production to verify virulence protein content. Immulon-2HB (Thermo Scientific) were coated with 100 μL of the vaccines of separate runs (vaccine A_ref_ -E) diluted to a final OD_590nm_ of 0.2. Plates were sealed and incubated overnight at room temperature. The next day, triplicate wells coated with each vaccine run were incubated with antibodies specific for six *B*. *pertussis* virulence proteins FHA (Mab 29E7), PRN (Mab Pem4), FIM2 (Mab 118E10), FIM3 (Mab 81H1), PT (Mab P8), Vag8 (Mab 14B4) and an antibody specific for band A LPS (Mab 88F3) or band B LPS (Mab BL-8) for 1 hour at 37°C. Binding of the antibodies was detected using an HRP-conjugated goat anti-mouse IgG in PBS containing 0.5% skim milk followed by incubation with peroxidase substrate (0.1 mg/mL TMB with 0.012% H_2_O_2_ in 0.11 M sodium acetate buffer [pH 5.5]) for 10 minutes. The reaction was stopped by addition of 100 μl 2 M H_2_SO_4_ to each well. The absorbance at 450nm was then measured using an ELISA reader (Bio-Tek). Heat-inactivated whole bacteria of the 509 strain that were either treated or not treated with formaldehyde were used to determine the effect of formaldehyde treatment on antibody recognition of target proteins.

### Cell lines and culture conditions

HEK-Blue cells stably transfected with human TLR4, MD-2 and CD14 (HB-hTLR4) or stably transfected with human TLR2 and CD14 (HB-hTLR2) were purchased from InvivoGen. As a control, the HEK-Blue Null1-cell line (HB-Null1) was used to determine the effect of endogenously expressed receptors. These cell lines express a secreted embryonic alkaline phosphatase (SEAP) reporter gene under the control of NF-κB-inducible promoter. HEK-293 cells stably transfected with murine TLR4, MD-2 and CD14 (HEK-mTLR4) were purchased from InvivoGen. To study the combinational effect of PRR, the human monocytic cell line MM6 was used [31]. All HEK cell lines were grown in DMEM and the MM6 cell line was grown in IMDM. Both media were supplemented with 10% heat-inactivated FCS, 100 U/mL penicillin, 100 μg/mL streptomycin, 300 ng/mL L-glutamine. In addition, media were supplemented with 1x HEK-Blue selection antibiotics and Normocin (HB-hTLR4 and HB-hTLR2), Zeocin and Normocin (HB-Null1), Blasticidin, HygroGold and Normocin (HEK-mTLR4) or 20 μM β-mercaptoethanol (MM6). All cells were cultivated at 37°C in a humidified atmosphere of 5% CO_2_.

### Generation and culture of moDC

For generation of moDC, peripheral blood from healthy donors was used. Donor blood was kindly provided by the internal blood donor system of the National Institute for Public Health and the Environment (RIVM) in the Netherlands. This study was conducted according to the principles expressed in the Declaration of Helsinki. All donors provided written informed consent for the collection of samples and subsequent analysis. The blood samples were processed anonymously. Peripheral blood mononuclear cells were isolated by density centrifugation on Lymphoprep (Nycomed) at 1000xg for 30 minutes. Cells were washed, harvested, and resuspended in PBS supplemented with 0.5% BSA and 2 mM EDTA. The cells were incubated with anti-CD14^+^ microbeads and CD14^+^ monocytes were isolated by magnetic sorting using MACS columns (Miltenyi Biotech). CD14^+^ positive cells were then cultured in 24-wells plates at 4 × 10^5^ cells/mL in IMDM supplemented with penicillin (100 U/mL), streptomycin (100 μg/mL), L-glutamine (300 ng/mL), 1% heat-inactivated FCS, human GM-CSF (500 U/mL), and human IL-4 (800 U/mL) for a total of 6 days.

### Stimulation of cell lines and moDC with wP vaccines and use of hTLR2 and hTLR4 blocking agents

HB-hTLR2, HB-hTLR4 and HB-Null1 cells were seeded in 96-well plates at 50000, 25000 and 50000 cells/well (96 well), respectively, and cultivated overnight. The HB-hTLR4, HB-hTLR2 and HB-Null1 cells were subsequently stimulated overnight with wP vaccines Aref—E at the indicated OD_590nm_. The activation of these cell lines was measured by mixing 20 μL of cell supernatant with 180 μL of QUANTI-Blue substrate™ followed by incubation at 37°C for two hours. The absorption at 649nm was measured using a microplate reader (Bio-Tek). MM6 cells were plated at 1.5 x 10^5^ cells/well (96-well plates), just prior to addition of wP vaccine Aref—E at indicated OD_590nm_ or the control stimulants described below. Activation of MM6 cells was assessed by measuring IL-6 and IL-12p40 secretion in culture supernatant using ELISA and the absorbance was measured at 450nm. As controls, HB-hTLR4, HB-hTLR2 and HB-Null1 and MM6 cells were stimulated with LPS-EC or PAM at indicated concentrations. Blocking of TLR4, TLR2 or both on HB-hTLR4 or MM6 cells was performed by incubating cells with LPS-RS (1 μg/mL), α-TLR2 antibody (0.5 μg/mL) or α-TLR2 antibody (0.5 μg/mL) and LPS-RS (1 μg/mL) for three hours. Subsequently, both cell lines were exposed to wP vaccines and control stimulants overnight and activation was measured by IL-6 and IL-12p40 secretion in the supernatant. In order to block TLR4 or TLR2 signalling or both on moDC, these cells were pre-incubated with LPS-RS (1 μg/mL), or α-TLR2 (0.5 μg/mL), or both TLR2 and TLR4 antagonists for three hours. Subsequently, the moDC were stimulated with wP vaccines A_ref_, C, E, LPS or PAM at the indicated concentration for two days. After stimulation, the presence of IL-12p40 in the supernatants of these cells was then measured using ELISA.

### Mass Spectrometry (MS) analysis of LPS

The LPS from 250 μl of vaccine preparations of *B*. *Pertussis* were extracted with hot phenol/water as described elsewhere [32]. LPS was purified further for mass spectrometry by using ZipTipC4 micropipette tips (Merck Millipore Ltd, Tullagreen, Carrigtwohill, Co. Cork, Ireland). Electrospray ionization mass spectrometry (ESI-MS) was performed on an LCQ Classic quadrupole ion trap mass spectrometer (Finnigan, San Jose, CA) in the negative-ion mode. Typically, from 5 to 10 μl of LPS in 50% (v/v) 2-propanol, 0.07 mM triethylammonium acetate pH 8.5 were infused into the mass spectrometer by static nanoelectrospray using gold-coated, pulled glass capillaries [33, 34]. The spray voltage was set to -2 kV and the capillary temperature to 200°C. Under these ionization conditions, no appreciable fragmentation of LPS was produced. Composition proposals for LPS molecular ions were based on the chemical structure of the LPS from *B*. *pertussis* reported previously [35].

### Microarray analysis

mRNA expression profiles of the *B*. *pertussis* bacteria harvested at different time points after sulfate addition were analysed using full genome *B*. *pertussis* DNA-microarrays according to the procedure described in detail elsewhere [12](Metz *et al*. submitted for publication). The data processing steps were done with the free statistical software R (http://www.r-project.org, R Foundation for Statistical Computing, Vienna, Austria), using an in-house developed script [12]. P-values for expression changes at any of the time points were calculated using a one-way ANOVA statistical analysis. The resulting p-values were then adjusted for multiple testing by calculating the false discovery rate (FDR). Maximal fold ratio (FR) values were expressed as the maximal/minimal normalized signal value between any of the time points. A p-value of 0.01 (FDR of 10%) was used to select genes whose gene-expression showed a statistically significant difference. To further select for biologically relevant effects, only statistically significant genes with a maximal FR above 1.25 were included in the final analysis.

### Gas chromatography of fatty acids in wP vaccines

The amount of fatty acids and the fatty acid composition in lipids within the wP vaccines was analysed using a modified gas chromatography method as described elsewhere [36]. The fatty acid methyl-esters were analysed based on their retention times compared to retentions times of the commercial standards. For quantification of hydroxy-fatty acids C14:0-3OH and C12:0-2OH were used as calibration standard and internal standard, respectively. For non-hydroxy-fatty acids C14:0 was used as standard and C15:0 was employed as internal standard.

### Statistical analysis

Data are presented as the mean ± the standard deviation of three independent determinations, unless mentioned otherwise. Unless mentioned otherwise, significant differences were analysed between the reference vaccine (A_ref_) and the other vaccines (B—E) using a Student’s t-test and considered significant when p<0.05.

## Results

### Modulation of the BvgASR system affects protein composition, but not LPS quantity of wP vaccines

**Fig 1:**
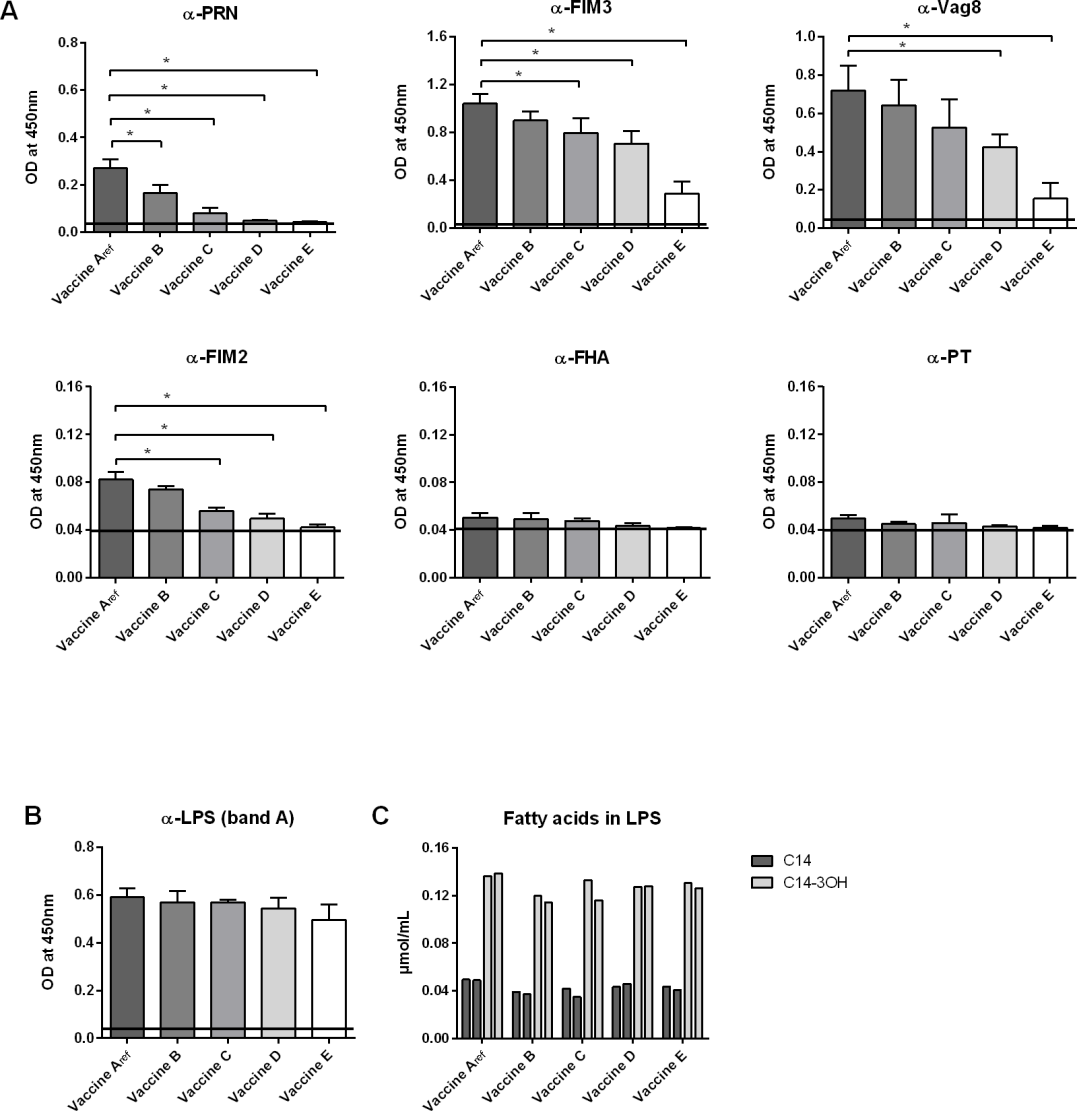
Bvg status of *B*. *pertussis* bacteria at time of harvest affects protein composition of the resulting wP vaccines. Amounts of proteins (A) and LPS (B) present in wP vaccines A_ref_, B, C, D, E (harvested 0, 2, 6, 12, 24 hours after the addition of sulfate, respectively) derived from three individual *B*. *pertussis* culture runs were measured by ELISA using specific monoclonal antibodies directed against individual proteins or LPS. (C) Fatty acid composition of vaccines A_ref_, B, C, D, and E analysed using a modified gas chromatography method (in duplicate). The black lines indicate the background levels measured in PBS only by ELISA. * = p < 0.05.

The relative amounts of six important virulence proteins in vaccine A_ref_—E, harvested 0–24 hours after the addition of sulfate, were determined using monoclonal antibodies to verify if the sulfate-induced suppression of the BvgASR system during the production process had resulted in differences in wP vaccine protein composition and thus quality (Fig 1A). PRN, FIM3 and Vag8 proteins were readily detectable in reference vaccine A_ref_, whereas FIM2 levels were clearly lower. In contrast, FHA and PT protein levels were at the lower limit of detection in all runs of each vaccine tested. Importantly, PRN, FIM3 and Vag8 protein levels decreased steadily over time and this decrease proved significant starting two (PRN), six (FIM3) and twelve hours (Vag8) after the addition of sulfate. Similarly, FIM2 protein levels were also significantly decreased six hours after the addition of sulfate. The levels of FIM2 and PRN were minimal and close to zero after 24 hours. These data clearly confirm that sulfate-mediated suppression of virulence during *B*. *pertussis* cultivation had resulted in the production of experimental wP vaccines of different protein compositions. In general, the levels of virulence proteins present in the wP vaccines coincided with the gradually decreasing potencies of these vaccines as determined in the *in vivo* Kendrick test (potency of vaccine A_ref_ was 7.0 IU/mL (95% interval 2 IU/mL and 27 IU/mL), the potency of vaccine C was 4.8 IU/mL (95% interval 2 IU and 10 IU) and the potency of vaccine E was 0.8 IU/mL (95% interval 0 IU/mL and 3 IU/mL) (Metz *et al*., submitted for publication)). According to the acceptance criteria of the European Pharmacopeia which specify a potency of at least 4 IU/mL and 95% interval with a lower limit of 2 IU/mL, the potencies of vaccine A and C were sufficient, whereas the potency of vaccine E was insufficient. Therefore, based on assessment of protein content and *in vivo* potency testing, we considered the qualities of vaccines A_ref_—E to range from good to poor.

The amount of LPS, another virulence factor of *B*. *pertussis* [15, 37], is not known to be regulated by the BvgASR system. In line with this, LPS was detected in all vaccines using an antibody specific for *B*. *pertussis* band A LPS and no significant differences were observed between vaccines A_ref_—E regarding their LPS content (Fig 1B). In addition, quantification of LPS in vaccine A_ref_—E by gas chromatography of LPS-specific fatty acids (non-hydroxy and hydroxy) revealed that there were no pronounced differences among the vaccines (Fig 1C). These results demonstrate that sulfate-mediated modulation of the BvgASR system did not affect the quantity of LPS in these wP vaccines.

Since formaldehyde treatment of the vaccines could have affected epitope recognition by the antibodies used in the ELISA, its effect on antibody recognition of virulence proteins was evaluated using *B*. *pertussis* strain 509 treated with or without formaldehyde (S1 Fig). Formaldehyde treatment had no effect on epitope recognition by FIM3- and Vag8-specific antibodies, while the detected levels of FIM2, FHA and PT were slightly lower. Surprisingly, detected PRN levels were higher after formaldehyde treatment. These results show that although formaldehyde treatment can affect the availability of epitopes for some of the antibodies, it does not seem to be responsible for the absence of an FHA- and PT-specific signal in vaccines Aref—E (Fig 1A). It is therefore likely that both proteins are absent or present at very low concentrations in the vaccine preparations rather than not detected.

### Quality of wP vaccines affects human TLR4 but not TLR2 signalling

**Fig 2:**
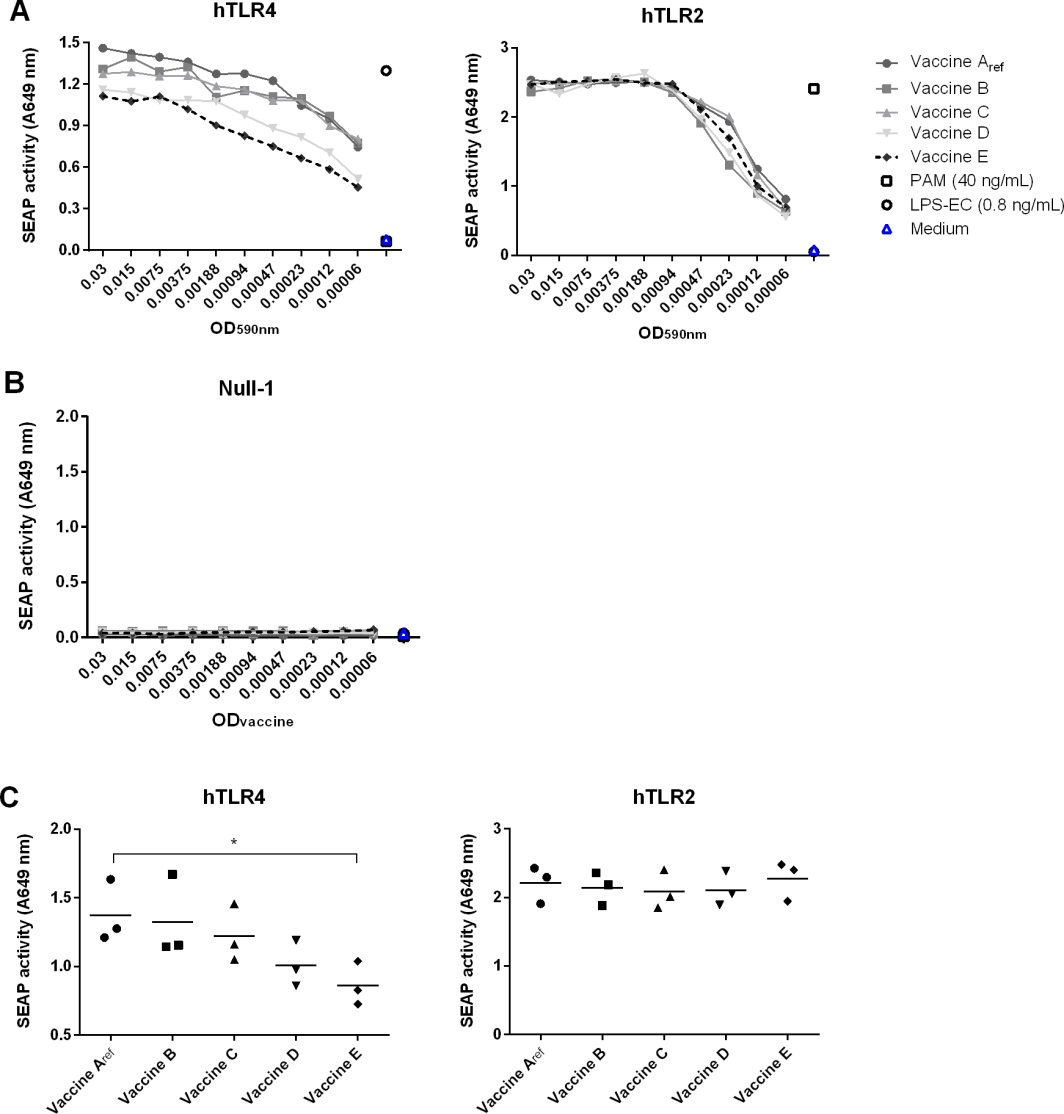
Activation of hTLR4- and hTLR2-mediated signalling by wP vaccines A_ref_—E. HB-hTLR2, HB-hTLR4 and HB-Null-1 cells were stimulated overnight with wP vaccines A_ref_, B, C, D, E, LPS-EC (0.8 ng/mL) or PAM (40 ng/mL). Shown is the SEAP activity in supernatants of HB-hTLR2, HB-hTLR4 (A) and HB-Null-1 (B) cells in response to 2-fold serial dilutions of the vaccines (representative responses are shown from one out of three independent experiments). (C) Shown is the SEAP activity of HB-hTLR2 and HB-hTLR4 cells in response to vaccines A_ref_, B, C, D and E at an OD_590nm_ of 0.00094. Each dot represents one value of three individually performed cell culture experiments. * = p < 0.05.

It is well established that *B*. *pertussis* can activate TLR4 and TLR2. However, while there is no evidence that *B*. *pertussis* can actively modulate TLR2 activation, it has been reported that *B*. *pertussis* is able to modify the structure of its LPS in a BvgASR dependent manner, thereby influencing host TLR4 signalling [26, 27]. Therefore, the capacity of wP vaccines A_ref_—E to activate human TLR4 (hTLR4) and TLR2 (hTLR2) was tested using HB-hTLR4 and HB-hTLR2 reporter cell lines. The vaccines induced a dose dependent production of SEAP through both hTLR4 and hTLR2 (Fig 2A). Responses were hTLR4 and hTLR2 specific since SEAP activity was not induced in the HB-Null1 control cells (Fig 2B). Importantly, vaccine E induced a consistently lower hTLR4 response than vaccines A_ref_, B, C and D for all shown dilutions (Fig 2A). The difference between vaccine A_ref_ and E proved significant for each of the four vaccine concentrations shown in three independent experiments (Fig 2C and S2A Fig). Significant differences in hTLR4 responses were not observed when the cells were stimulated with wP vaccines at higher or lower ODs. In contrast, no consistent differences in hTLR2 activation were found between vaccines A_ref_—E (Fig 2A and 2C), although there was some variation at indicated ODs (S2B Fig). Together, these results demonstrate that the Bvg status of the bacteria at the time of harvest influenced the capacity of the vaccines to induce hTLR4 signalling, while leaving hTLR2 signalling capacity largely unaffected.

### wP vaccines of varying quality differ in their capacity to activate APC, primarily in a human TLR4-dependent manner

**Fig 3:**
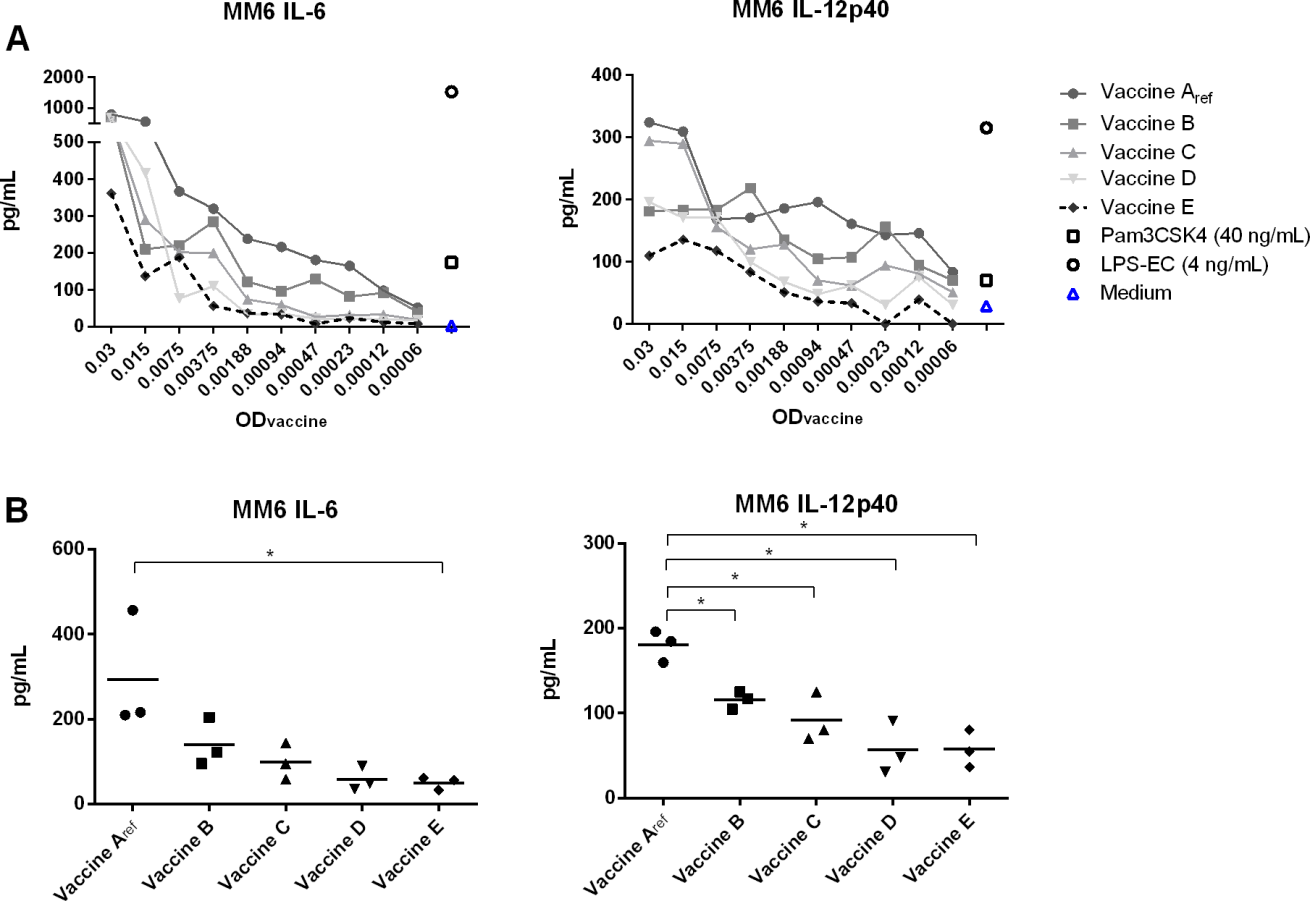
Secretion of IL-6 and IL-12p40 by MM6 cells stimulated with wP vaccines A_ref_—E. MM6 cells were stimulated overnight with vaccines A_ref_, B, C, D, E, LPS-EC (4 ng/mL) or PAM (40 ng/mL). Subsequently, IL-6 and IL-12p40 secretion was measured in culture supernatants. (A) Shown is the response of MM6 cells to 2-fold serial dilutions of vaccine A_ref_, B, C, D and E (representative responses are shown from one out of three independent experiments). (B) Response of MM6 cells to vaccines A_ref_, B, C, D and E at an OD_590nm_ of 0.00094. Each dot represents one value of three individually performed cell culture experiments. * = p < 0.05.

In order to investigate if wP vaccines A_ref_—E also differed in their capabilities to activate innate immune cells expressing several different TLRs, vaccine-induced activation of MM6 cells and moDC was studied, as well as the contribution of hTLR2 and hTLR4 signalling to the activation of these cell types. MM6 is a human monocytic cell line that expresses both TLR2 and TLR4 [38, 39] and responded to purified agonists for these receptors as well as our wP vaccines (Fig 3A) [28]. Vaccine E induced consistently lower IL-6 and IL-12p40 secretion by MM6 cells for all indicated ODs compared with vaccine A_ref_ (Fig 3A), while vaccines B, C and D induced secretion of intermediate amounts of these cytokines. Importantly, these differences between vaccine A_ref_ and E were significant at indicated ODs in three independent experiments (Fig 3B and S2C and S2D Fig). This indicates that also activation of MM6 cells by wP vaccines was affected by the bacterial Bvg status at the time of harvest during vaccine production.

**Fig 4:**
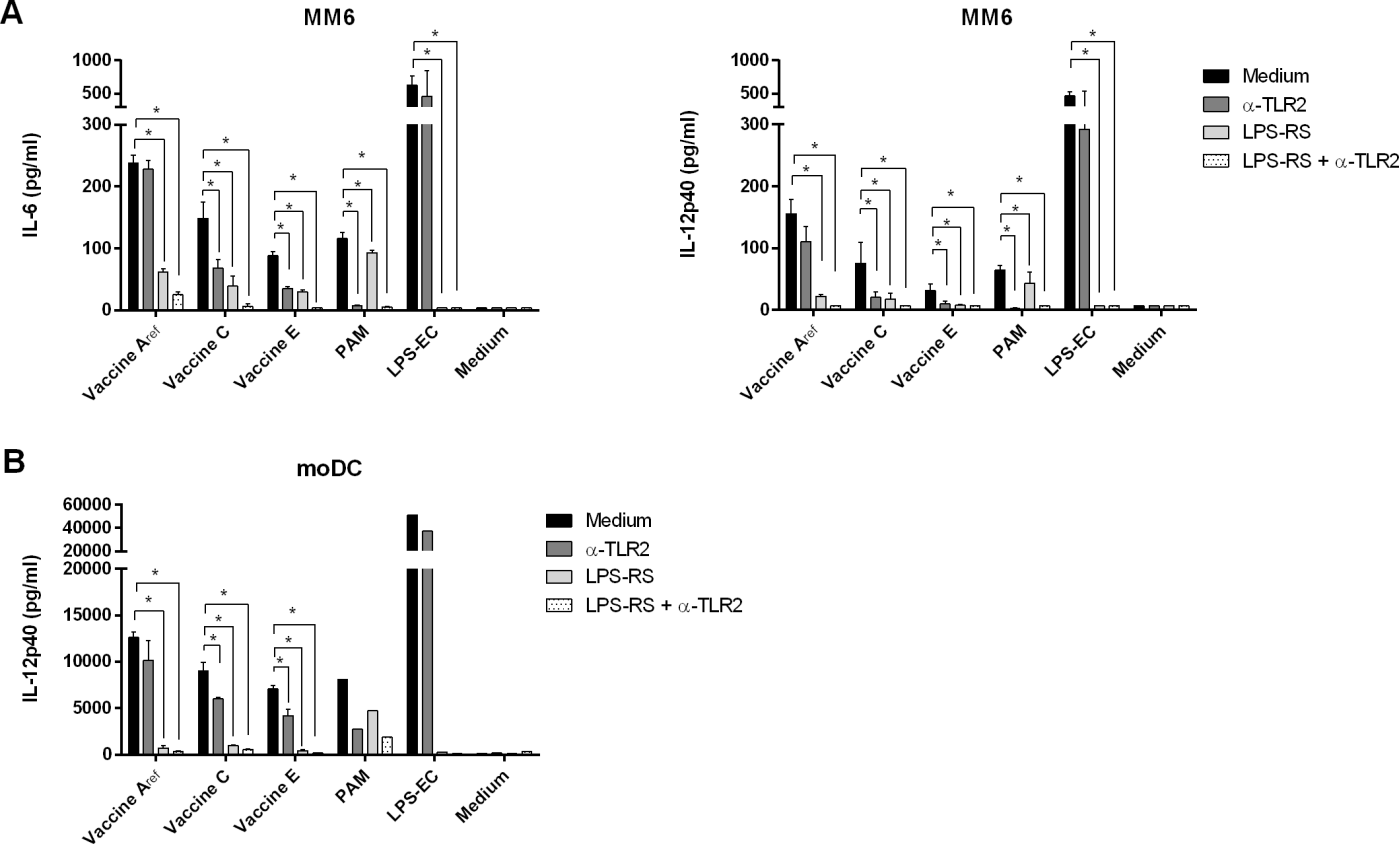
Activation of MM6 cells and moDC by wP vaccines is primarily mediated by hTLR4 signalling. MM6 cells and moDC were pre-treated for 3 hours with the TLR4 antagonist LPS-RS (1 μg/mL), a blocking antibody against human TLR2 (0.5 μg/mL) or a combination of both. Subsequently, MM6 cells were stimulated with wP vaccine A_ref_, C, E (OD_590nm_ of 0.00094), PAM (100 ng/mL), LPS-EC (4 ng/mL) or medium overnight. Similarly, moDC were stimulated for 2 days with vaccine A_ref_, C and E (OD_590nm_ of 0.00047), LPS-EC (100 ng/mL), PAM (1 μg/mL) or only medium. (A) Secretion of IL-6 and IL-12p40 by MM6 cells measured in culture supernatants (one experiment out of two experiments with similar results is shown). (B) Secretion of IL-12p40 by moDC measured in culture supernatants (one experiment out of two experiments with similar results is shown). * = p < 0.05.

Since the vaccines derived from *B*. *pertussis* bacteria harvested after the addition of sulfate (vaccine B-E) displayed a clear trend towards lower hTLR4 activation than our reference vaccine derived from this bacterial culture before sulfate was added (vaccine A_ref_), we wanted to gain more insight into the relative contribution of hTLR4- and hTLR2-mediated signalling to overall vaccine-induced activation of APC. To study this, MM6 cells were pre-incubated with a constant concentration of the TLR4 antagonist LPS-RS, a TLR2 blocking antibody or both. Blocking of hTLR2 on MM6 cells significantly decreased the IL-6 and IL-12p40 secretion in response to vaccine C and E (Fig 4A). However, hTLR2 blocking had a minor effect on secretion responses to vaccine A_ref_, while hTLR4 blocking significantly decreased the MM6 cell response to all wP vaccines tested. When both hTLR2 and hTLR4 were blocked, the MM6 cell response to the wP vaccines became marginal. These data suggest that hTLR4-mediated signalling was the main contributor to MM6 activation by vaccine A_ref_ with no or a limited role for hTLR2. The relative contribution of hTLR4 to activation of these cells by vaccines of lower quality (C and E) deceased gradually, while the relative contribution of hTLR2 increased and was significant for vaccine C and E.

Since dendritic cells (DC) are professional APC that direct adaptive immune responses, the response of moDC to vaccines A_ref_, C, and E was investigated next. MoDC were pre-incubated with LPS-RS, α-TLR2 blocking antibody or both antagonists and stimulated with LPS, PAM and the wP vaccines A_ref_, C and E. Within the performed experiments, the α-TLR2 antibody largely, but not completely inhibited the response to the TLR2 ligand PAM, most likely caused by incomplete inhibition of all hTLR2 receptors (Fig 4B). In a similar way as observed with MM6 cells, blocking of hTLR2 on moDC significantly decreased IL-12p40 secretion in response to vaccine C and E, but not to vaccine A_ref_, while hTLR4 blocking significantly reduced the response to all wP vaccines tested. Simultaneous blockade of hTLR2 and hTLR4 in both wP stimulated MM6 and moDC resulted in a response close to the lower limit of detection of the used ELISAs, suggesting only TLR2 and TLR4 ligands rather than ligands for other PRR play a role in wP activation of APC. In general, the relative contribution of hTLR4 signalling to the activation of both cell types was higher compared with hTLR2 signalling. In addition, this effect was clearly more pronounced when vaccine A_ref_ was used as a stimulant and could explain the differences in APC activation induced by these wP vaccines of varying quality.

### wP vaccines produced under Bvg-modulating conditions contain structurally different LPS molecules

**Fig 5:**
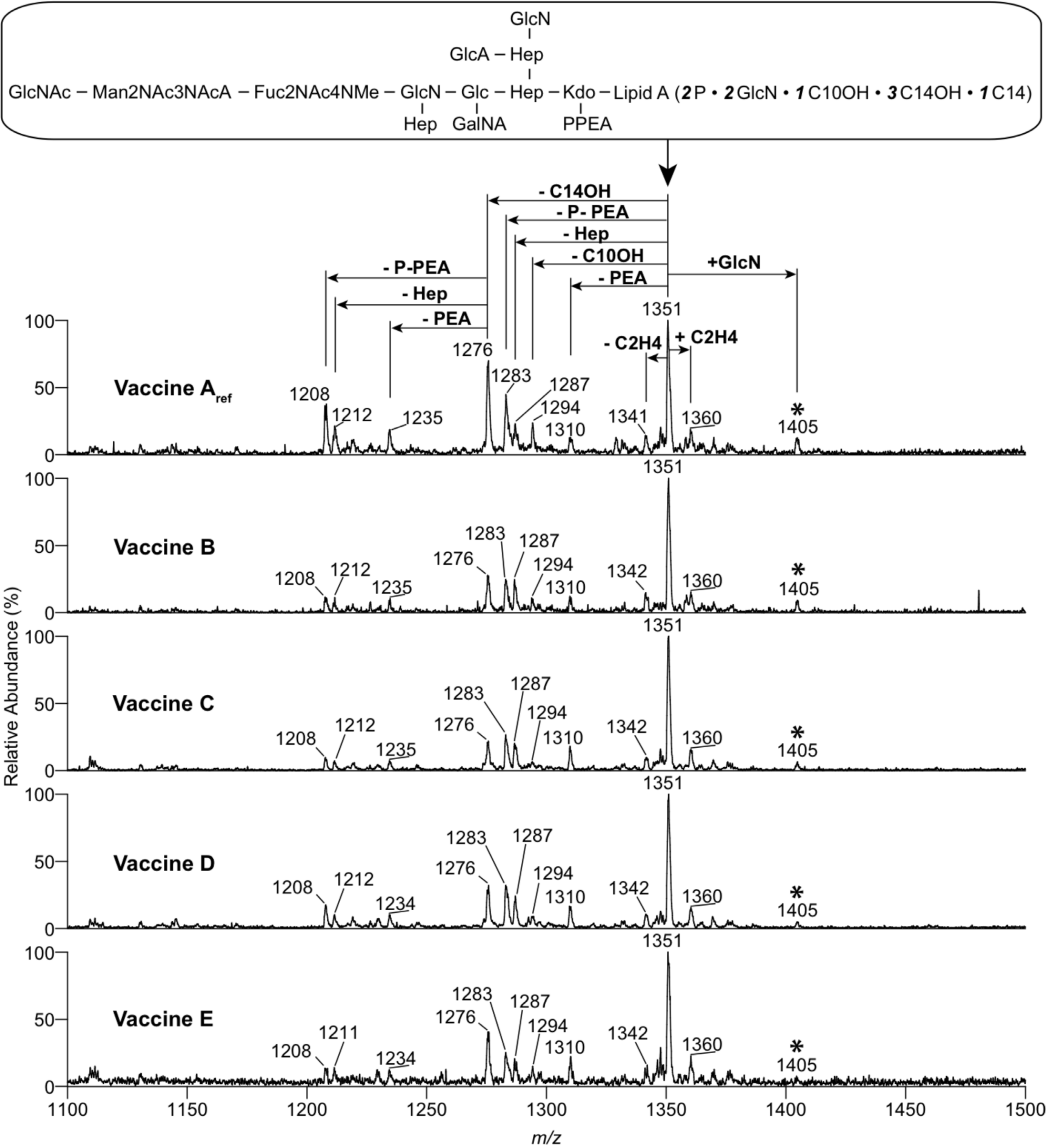
Negative-ion ESI mass spectra of LPS isolated from wP vaccine preparations A_ref_—E. The triply charged (M-3H)3- molecular ion regions of the mass spectra of LPS from vaccine preparations A_ref_, B, C, D and E are shown. The box on top of the mass spectra contains a simplified representation of the chemical structure of the LPS from *B*. *pertussis* reported previously [35]. This structure has been assigned to the ion of m/z 1351. The ions highlighted by an asterisk correspond to LPS with a glucosamine substitution of the lipid A phosphate. Abbreviations: Kdo, 3-deoxy-D-manno-oct-2-ulosonic acid; Hep, L-glycero-D-manno-heptose; Glc, glucose; GlcN, glucosamine; GlcNAc, N-acetyl glucosamine; Fuc2NAc4NMe, 2-acetamido-4-N-methyl-2,4,6-deoxy-galactose; GalNA, galactosaminuronic acid; GlcA, glucuronic acid; Man2NAc3NAcA, 2-acetamido-3-acetamido-2,3-dideoxy-mannuronic acid; PPEA, pyrophosphoethanolamine; P, phosphate; C14OH, 3-hydroxy-tetradecanoic acid; C14, tetradecanoic acid; C10OH, 3-hydroxy-decanoic acid.

Since the different hTLR4 signalling capacities of vaccines Aref—E cannot be explained by differences in LPS quantity, we hypothesised that this might be the result of variations in the structure of the LPS molecules present in vaccines A_ref_—E. To investigate this, LPS from the vaccine preparations was isolated and analysed by negative-ion ESI-MS. Mass spectrometry analysis showed that all vaccines mainly contained penta-acylated LPS carrying a branched dodecasaccharide chain (*m/z* 1351, Fig 5) of the same composition as that reported previously for the main species (band A LPS) of LPS from *B*. *pertussis* [35]. Similarly, minor LPS species were present in all vaccine preparations corresponding to tetra-acylated LPS lacking a 3-hydroxy-tetradecanoic acid (m/z 1276) or 3-hydroxy-decanoic acid (m/z 1294) as well as tetra-acylated and penta-acylated LPS species that lost a heptose (m/z 1212 and 1287, respectively), a phosphoethanolamine (m/z 1235 and 1310, respectively) or a pyrophosphoethanolamine group (m/z 1208 and 1283, respectively) from the dodecasaccharide core (Fig 5). However, when comparing the LPS spectra derived from vaccines A_ref_—E, the peak corresponding to LPS carrying a GlcN substitution of lipid A phosphate (m/z 1405) gradually decreased from vaccine A_ref_ toward vaccine E (Fig 5). Since the wP vaccines did not differ in LPS amount (Fig 1B and 1C), it is likely that the presence of the GlcN modification on the lipid A of the LPS molecules in vaccine A_ref_ and the gradual decrease of this modification on the lipid A from vaccines B—E is responsible for the observed different capacities of the vaccines to induce hTLR4 signalling and APC activation.

Previously, it has been shown that GlcN modification of *B*. *pertussis* lipid A specifically affects human TLR4 signalling, while this modification has no effect on murine TLR4 (mTLR4) signalling [24]. Therefore, the capacity of vaccines A_ref_, C and E to bind and activate murine TLR4 was tested using a HEK-mTLR4 reporter cell line. All vaccines induced a dose dependent production of IL-8, but no consistent differences between the mTLR4 responses induced by vaccine A_ref_, C and E were observed (S3 Fig). Additional evidence for the presence of GlcN modifications comes from an experiment in which the vaccines were incubated with the antibiotic polymyxin B. GlcN modification of LPS is a known mechanism used by *Bordetella* bacteria to confer resistance to neutralisation with the antibiotic polymyxin B [40]. Incubating the wP vaccines with this antibiotic before stimulating the MM6 cells, demonstrated that vaccine A_ref_ was resistant to polymyxin B neutralisation, while vaccine E was neutralised and no longer induced MM6 cell activation (S4 Fig). Both the absence of a difference in mTLR4 activation and the association between the GlcN modification and the resistance to polymyxin B, provide further evidence that the variations in GlcN substitution of the LPS molecules present in these vaccines are a main determinant for the observed differences in activation of hTLR4-expressing cells.

### Prolonged culturing of *B*. *pertussis* in the presence of sulfate is associated with differential expression of genes encoding LPS modifying enzymes

**Fig 6:**
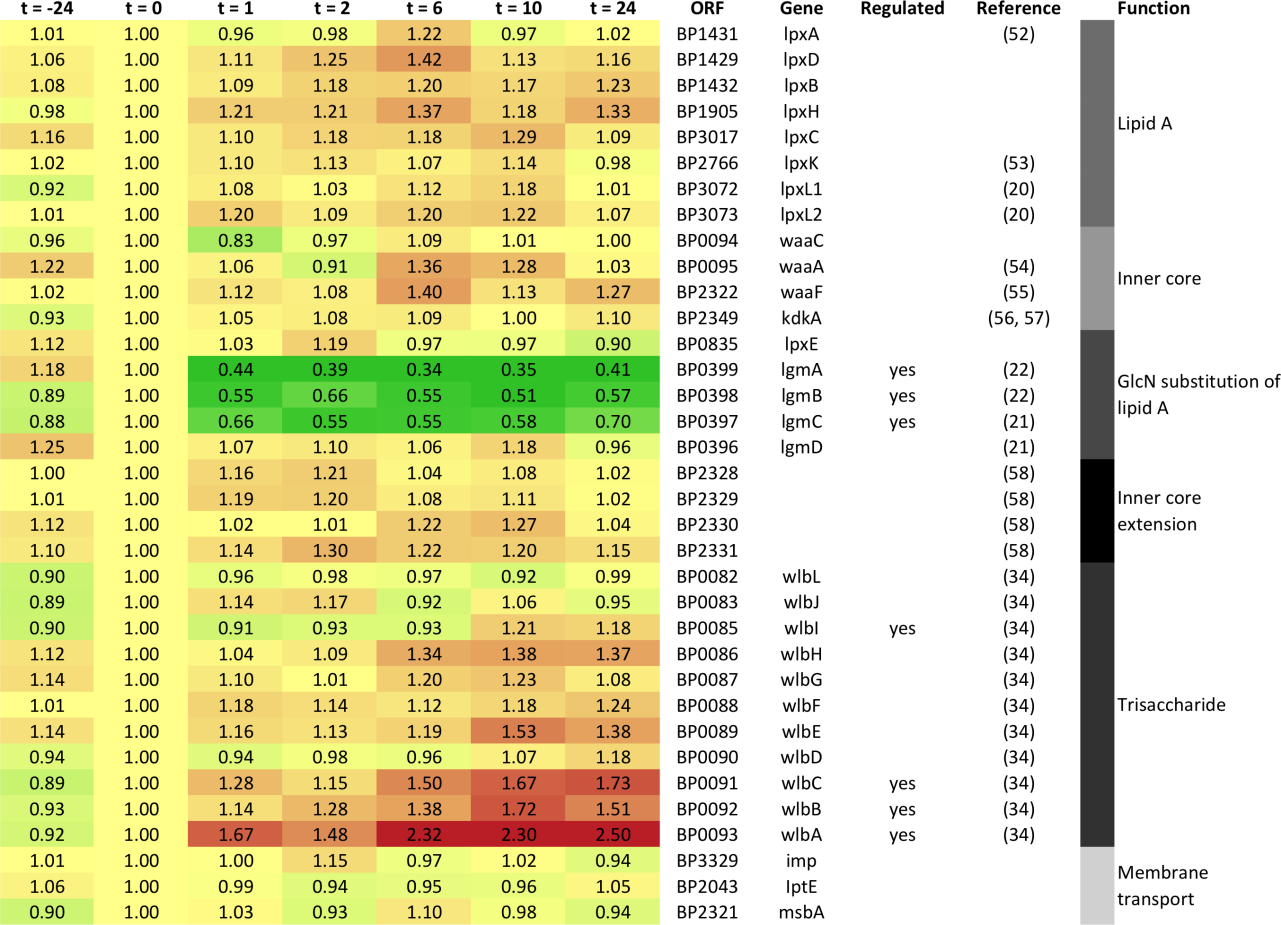
Expression of genes associated with LPS synthesis and modification in *B*. *pertussis* in response to sulfate exposure. Relative gene expression of a panel of 35 genes associated with LPS synthesis or modification in *B*. *pertussis* [25–27, 41, 44–50]. Each row represents the relative transcript abundance of a single gene in *B*. *pertussis* bacteria harvested before and at different time points after sulfate addition to the growth medium. Column names (t = -24 –t = 24) correspond to the time points after the addition of sulfate at which bacteria were harvested (0 = vaccine A_ref_, 2h = vaccine B, 6h = vaccine C, 12h = vaccine D, 24h = E). The colour scale indicates gene regulation ranging from strong downregulation (dark green), to no regulation (yellow), and strong upregulation (dark red). Genes for which the expression changed significantly throughout the production process are marked with an asterisk (p < 0.05).

Because of the differences found in the LPS structures present in vaccines A_ref_—E and the clear differential hTLR4 signalling capacities of these wP vaccines, we wondered whether differential expression of genes involved in LPS modification would form the basis for these findings. Therefore, the gene expression profiles of a panel of 35 *B*. *pertussis* genes, known to be associated with synthesis or modification of LPS were analysed. This data set was derived from a whole-genome microarray experiment performed with RNA samples isolated from a sample of the bacterial culture just before the production of vaccines A_ref_—E (Metz *et al*., submitted for publication). The expression profiles revealed that addition of sulfate did not alter the expression of most of these genes (Fig 6). However, the gene expression profiles revealed that sulfate addition induced significantly enhanced expression of four genes that are part of the *wlb* gene cluster (*wlbA*, *wlbB*, *wlbC* and *wlbI*). This locus is associated with the addition of a trisaccharide moiety to the core structure of *B*. *pertussis* LPS, producing an LPS form known as band A LPS [41–43]. To investigate if this had any effect on the ratio of band A and B LPS in vaccines Aref—E, we determined the relative amounts of these LPS species using band A or band B specific antibodies (S5 Fig). Interestingly, band B LPS proved to be nearly undetectable in all vaccines, whereas clear differences in band A LPS amounts among the vaccines were not observed. As significant changes in the presence of the band A-specific trisaccharide were also not found by MS analysis of the LPS molecules present in vaccines Aref—E (Fig 4), we cannot present any evidence for structural alterations of the LPS in the different vaccines, caused by sulfate-induced changes in the expression of the four *wlb* cluster genes. Most importantly, the expression of the *lgmA*, *lgmB* and *lgmC* genes, known to be responsible for GlcN modification of lipid A in *B*. *pertussis* [26], was significantly reduced following growth in sulfate-containing medium. This strongly suggests that changes in the gene expression of these enzymes are responsible for the gradual decrease in GlcN modification of lipid A that was observed in vaccines B—E (Fig 5).

## Discussion

Differences in the culture conditions used during wP vaccine production can be sensed by the BvgASR regulatory system of *Bordetella pertussis* [4, 15] and can thereby influence the expression of proteins involved in virulence. Several studies have linked the quality of wP vaccines to the presence of these virulence-associated proteins [16, 17, 51]. Recently, we have shown that moDC and MM6 cells represent suitable platforms to measure differences in the innate immune activation capacities of experimental wP vaccines of varying quality (A_ref_—E) [28], that were prepared by deliberate manipulation of the bacterial BvgASR system during the production process (Metz *et al*., submitted for publication). In the present study, we showed that the differences in APC activation are largely caused by the distinct capacities of these vaccines to activate hTLR4. Additionally, we present evidence strongly suggesting that the observed differences in hTLR4 activation induced by our wP vaccines are directly linked to the amount of GlcN-modified LPS molecules present in these vaccines, which in turn is dependent on BvgASR-controlled expression of the *lgmA*, *lgmB* and *lgmC* genes. These findings demonstrate that wP vaccine quality is connected to their capacity to activate human TLR4 and suggest that this association could serve as a useful parameter for *in vitro* assessment of wP vaccine quality.

Sulfate-mediated repression of the BvgASR system is known to impair the expression of *B*. *pertussis* virulence factors [52] and this method was therefore used within this study to mimic potential problems that could occur during vaccine production, such as nutrient limitation at the end of bacterial cultivation [12]. This way, a panel of five experimental wP vaccines of varying quality (A_ref_—E) was produced by culturing *B*. *pertussis* bacteria either in the absence or presence of sulfate for different time spans. The protein composition of these vaccines was analysed in detail using ELISA and mass spectrometry immediately after vaccine production and this clearly confirmed that suppression of BvgASR-controlled gene expression in *B*. *pertussis* had resulted in vaccine products of different protein contents. These differences were also reflected in the potency of these vaccines, as determined for vaccines A_ref_, C and E in the intracerebral challenge test (Metz *et al*., submitted for publication). Using vaccines A_ref_—E, in this study the effect of culture condition-induced changes in Bvg phase on various vaccine characteristics was investigated. To verify previous results on the protein composition of these vaccines and to show that the vaccines were still qualitatively different, we quantified the amount of several virulence antigens in these products by ELISA. The analysed antigens are considered to be important for the induction of protective antibody and T cell responses (FHA, PRN, FIM2, FIM3, PT and Vag8) [53–56]. Although the results in this study confirmed the earlier data, showing that wP vaccines A_ref_—E differ in antigen content, there are some slight differences between the results in both studies. The ELISA analysis described in this study, detected relatively lower concentrations of FIM2 and FHA in the vaccines compared to those measured previously by Metz *et al*., using both ELISA and mass spectrometry. This discrepancy between both analyses might be due to the use of different antibodies or the inherently higher sensitivity of a method such as mass spectrometry. In addition, the structure and stability of FIM2 and FHA may have changed since the first ELISA quantification, as the first ELISA was performed on heat inactivated, but not formaldehyde treated bacteria (Metz *et al*., submitted for publication), whereas the products analysed in the current ELISA were 1.5 years older and were both treated with formaldehyde and heat inactivated. Nevertheless, overall our data showed that wP vaccines (A_ref_—E) contained decreasing amounts of the virulence proteins FIM2, FIM3, Vag8 and PRN (Fig 1A), similar as described in Metz *et al*., thereby confirming the previous conclusion that vaccines (A_ref_—E) are qualitatively different, ranging from good (A_ref_) to poor (E).

Importantly, adaptive immune responses not only depend on a vaccine’s antigen composition, but also on the vaccines capacity to induce activation of APC, since these cells initiate and direct the adaptive immune response [19]. Activation of APC depends on the recognition of pathogen-associated molecular patterns by PRR. It is well known that wP vaccines induce APC activation through TLR2 and TLR4 [20] leading to NF-κB dependent secretion of cytokines. The contribution of TLR4 and TLR2 to wP vaccine-induced responses has been demonstrated *in vivo* in mice, where TLR4 contributed to the early innate immune response as well as to subsequent antibody and T cell responses, while TLR2 did not [22]. In our *in vitro* assays, we observed that the modulation of the Bvg system by sulfate had resulted in a gradual decrease in MM6 and HB-hTLR4 activation capacities when comparing responses to vaccines A_ref_—E (Figs 2 and 3). In addition, the differences in activation of MM6 cells and moDC between the vaccines mainly disappeared after blocking of TLR4 on the surface of these cells (Fig 4). In contrast, we found no indication that the hTLR2-activating capacities of vaccines A_ref_—E had been affected by sulfate addition during vaccine production (Fig 2), although activation of hTLR2 contributed to the innate immune cell response induced by wP vaccines C and E, but did not significant contribute to the response to vaccine A (Fig 4). A recent report identified several lipoproteins in *B*. *pertussis* and two of them were shown to be specific TLR2 ligands [21]. Thus far, there is no evidence for Bvg-dependent regulation of lipoprotein expression in *B*. *pertussis*, which is in line with our TLR2-activation results. Together, these results indicate that TLR4-mediated signalling was primarily responsible for the observed differences in responses of MM6 cells and moDC to vaccines A_ref_—E, whereas activation of TLR2 did not substantially contribute to these differences.

The major role of TLR4 signalling in MM6 cells and moDC activation by our wP vaccines was not unexpected, since mTLR4 is known to be involved in immune responses and protection against *B*. *pertussis* in mice [22, 23] and LPS of *B*. *pertussis* is known to induce activation of hTLR4 *in vitro* [57]. However, the observed differences in hTLR4 activation capacities of vaccines A_ref_—E were somewhat surprising, as no variation in LPS quantity between these vaccines was detected (Fig 1B and 1C). This implied that not LPS quantity, but LPS structure could be the reason for the different TLR4-activating abilities of vaccine A_ref_—E. Several studies have shown that *B*. *pertussis* can modify its lipid A by substituting the phosphate groups with GlcN in a BvgASR-controlled manner, resulting in enhanced hTLR4 activation [24]. In line with these studies, we demonstrated that LPS molecules derived from our good quality reference vaccine (A_ref_), produced from Bvg^+^ phase bacteria, were substituted with GlcN to the highest degree, while the LPS molecules isolated from vaccines B—E, produced from bacteria cultured in the presence of sulfate, displayed a gradual decrease in GlcN substitution (Fig 5). In contrast to previous studies, these changes in GlcN modifications were not the result of an introduced mutation in the *bvgS* or *lgmB* genes [24, 27], but induced by culturing *B*. *pertussis* in the presence of sulfate. We confirmed that sulfate induced changes in the expression of *lgmA*, *lgmB* and *lgmC* genes (Fig 6), known to be responsible for GlcN modification of lipid A in *B*. *pertussis* [26]. Though the LPS molecules substituted with GlcN represented a minor LPS species, this was the only species for which the amount consistently corresponded with the culture time in the presence of sulfate. Moreover, Shah *et al*. showed that GlcN-modification of LPS had a very strong effect on hTLR4 activation [24]. Since LPS quantity did not differ between our wP vaccines, it is likely that Bvg^+^ phase-dependent GlcN modification was responsible for the differences in hTLR4 signalling and APC activation induced by these vaccines, although we cannot completely exclude that other minor LPS species played a role in this as well.

Another remarkable characteristic of GlcN-modified LPS is that it confers resistance to neutralisation by the antibiotic polymyxin B, as demonstrated for *B*. *pertussis* [40] and *B*. *bronchiseptica* LPS [58]. Similar LPS modifications have been found in *E*. *coli* and *S*. *typhimurium* [59]. Interestingly, activation of MM6 cells by vaccine A_ref_, proved to be unaffected or even enhanced by polymyxin B addition, whereas vaccine E, derived from bacteria cultured in the presence of sulfate for 24 hours, was neutralised by polymyxin B and no longer induced hTLR4 signalling (S4 Fig). This observation suggests that polymyxin B specifically bound to LPS molecules without GlcN modification, within vaccine E leading to reduced activation of hTLR4, while it might have promoted the possibility of minor GlcN modified LPS species in vaccine A_ref_ to activate hTLR4. These data provide indirect evidence for the presence of GlcN-modified lipid A in wP vaccines A_ref_ and its absence in vaccine E.

The *wlb* gene cluster is responsible for the addition of a trisaccharide moiety to the core structure of *B*. *pertussis* LPS, thereby allowing the formation of band A LPS. In contrast, band B LPS does not carry a trisaccharide on its core structure [41, 43]. The expression of the *wlb* cluster has been shown to influence the colonization of *B*. *pertussis* in the lungs and trachea of mice [42]. Surprisingly, the expression of four of the genes of the *wlb* cluster, *wlbA*, *wlbB*, *wlbC* and *wlbH*, was significantly upregulated by bacterial growth in the presence of sulfate. Nevertheless, this did not alter the relative amounts of band A or B LPS in vaccines A_ref_—E (S5 Fig). This apparent discrepancy may be attributed to the relatively high amount of band A LPS already present in wP vaccine A_ref_. In addition, it may be possible that the trisaccharide moiety is synthesized in excess, but finally not attached to the LPS molecules.

A recent study demonstrated that the *lgmA*, *lgmB* and *lgmC* genes encode the enzymes that are required for the modification of *B*. *pertussis* lipid A with GlcN, while the enzyme encoded by the *lgmD* gene in the same cluster proved not essential [26]. Here, we showed that the *lgmA*, *lgmB and lgmC* genes were the major *B*. *pertussis* genes involved in LPS synthesis or modification of which the expression was significantly reduced by the addition of sulfate (Fig 6). This confirms that the transcription of these genes is indeed controlled by the BvgASR system [27] and provides an explanation for the steadily lower amounts of GlcN-modified LPS that were detected in those vaccines that were produced from bacteria cultured in the presence of sulfate for prolonged periods of time. Changes in *lgmA* and *lgmB* expression have also been described after culturing *B*. *pertussis* in the presence of only 5 mM sulfate, suggesting that the expression of both genes can also shift in response to minor environmental changes [54].

Our results demonstrate that suppression of the BvgASR system of *B*. *pertussis* bacteria in the process of wP production leads to a time-dependent gradual reduction in the amount of virulence proteins present in the resulting vaccines. However, this not only affects protein composition, but can also lead to specific structural alterations of the LPS molecules in the wP vaccines, leading to differences in hTLR4 signalling capacity. This study highlights the need for monitoring of the production process of whole-cell pertussis vaccines and provides examples of *in vitro* cell-based and physico-chemical tools, such as HB-hTLR4, MM6 and moDC cell systems, and mass spectrometry of LPS structure, that can be used for this purpose. Nevertheless, comprehensive validation experiments will be necessary to implement these tools. Furthermore, these data provide a scientific explanation for our recent proof-of-principle study showing that moDC and MM6 cells represent suitable platforms for *in vitro* monitoring of the consistency of the quality of wP vaccine production [28], thereby paving the road toward the development of suitable *in vitro* methods to assess the quality of these vaccines in the future.

## Supporting Information

S1 Fig Effect of formaldehyde treatment on detection of *B*. *pertussis* proteins by monoclonal antibodies. Amounts of proteins present in whole bacteria of strain 509 either treated with formaldehyde (50 mM) or not, were measured in an ELISA, using specific monoclonal antibodies directed against individual proteins. * = p < 0.05.
(TIF)

S2 Fig Activation of hTLR4- and hTLR2-mediated signalling by wP vaccines A_ref_—E at OD_590nm_ 0.00047–0.00012. HB-hTLR2, HB-hTLR4 and MM6 cells were stimulated overnight with wP vaccines A_ref_, B, C. D, E at an OD_590nm_ of 0.00047, 0.00023, 0.00012. Vaccine-induced SEAP secretion (HB-hTLR2 and HB-hTLR4) or IL-6 and Il-12 secretion (MM6 cells) was measured in culture supernatants. Each dot represents one value of three individually performed cell culture experiments. * = p < 0.05.
(TIF)

S3 Fig Activation of mTLR4 signalling by wP vaccines A_ref_, C and E. HEK-mTLR4 cells were stimulated overnight with wP vaccines A_ref_, C, E, or LPS-EC (200 ng/mL). NF-κB activity was measured by the secretion of IL-8 in the supernatants in response to 4-fold serial dilutions of the vaccines (representative responses are shown from one out of three independent experiments).
(TIF)

S4 Fig Neutralisation of *B*. *pertussis* LPS in vaccine A_ref_ and E by polymyxin B. Vaccines A_ref_ and E (OD_590nm_ 0.06) and LPS-EC (100 ng/mL) were pre-treated with varying concentrations of polymyxin B for 2 hours at 37°C. These solutions were used to stimulate the MM6 cells overnight. Activation of the MM6 cells was determined by assessing the amounts of IL-6 secreted into the supernatant using an ELISA (responses from one experiment out of two independent experiments with similar results are shown).
(TIF)

S5 Fig Effect of the BvgASR status of *B*. *pertussis* bacteria on band A and band B LPS within the wP vaccines. Amounts of band-A and band-B LPS in the pooled vaccine preparations A_ref_, B, C, D, E measured by ELISA (OD_590nm_ 0.2), using specific monoclonal antibodies against band-A and band-B. Inactivated bacteria of *B*. *pertussis* strain 0134 (OD_590nm_ 0.28) served as positive controls.
(TIF)

## References

[1] Libster R, Edwards KM. Re-emergence of pertussis: what are the solutions? Expert review of vaccines. 2012;11(11):1331–46. 10.1586/erv.12.118 . 23249233

[2] Mattoo S, Cherry JD. Molecular pathogenesis, epidemiology, and clinical manifestations of respiratory infections due to Bordetella pertussis and other Bordetella subspecies. Clinical microbiology reviews. 2005;18(2):326–82. 10.1128/CMR.18.2.326-382.2005 15831828 PMC1082800

[3] Sheridan SL, Ware RS, Grimwood K, Lambert SB. Number and order of whole cell pertussis vaccines in infancy and disease protection. Jama. 2012;308(5):454–6. 10.1001/jama.2012.6364 . 22851107

[4] Witt MA, Arias L, Katz PH, Truong ET, Witt DJ. Reduced risk of pertussis among persons ever vaccinated with whole cell pertussis vaccine compared to recipients of acellular pertussis vaccines in a large US cohort. Clinical infectious diseases: an official publication of the Infectious Diseases Society of America. 2013;56(9):1248–54. 10.1093/cid/cit046 . 23487373

[5] WHO. Pertussis vaccines. Wkly Epidemiol Rec. 2014;21(89):230–3.

[6] Liko J, Robison SG, Cieslak PR. Priming with whole-cell versus acellular pertussis vaccine. The New England journal of medicine. 2013;368(6):581–2. 10.1056/NEJMc1212006 . 23388023

[7] Xing D, Das RG, O'Neill T, Corbel M, Dellepiane N, Milstien J. Laboratory testing of whole cell pertussis vaccine: a WHO proficiency study using the Kendrick test. Vaccine. 2001;20(3–4):342–51. . 11672896 10.1016/s0264-410x(01)00372-3

[8] WHO. Recommendations for whole-cell pertussis vaccine. WHO Technical Report Series 2007;941, Geneva, Switzerland:301–33.

[9] Pharmacopeia E. Assay of pertussis vaccine (whole cell). 2013;8th edition:242.

[10] van Straaten-van de Kappelle I, van der Gun JW, Marsman FR, Hendriksen CF, van de Donk HJ. Collaborative study on test systems to assess toxicity of whole cell pertussis vaccine. Biologicals: journal of the International Association of Biological Standardization. 1997;25(1):41–57. 10.1006/biol.1996.0059 . 9167008

[11] Hendriksen CF, Steen B. Refinement of vaccine potency testing with the use of humane endpoints. ILAR journal / National Research Council, Institute of Laboratory Animal Resources. 2000;41(2):105–13. . 11417495 10.1093/ilar.41.2.105

[12] van de Waterbeemd B, Streefland M, Pennings J, van der Pol L, Beuvery C, Tramper J, et al Gene-expression-based quality scores indicate optimal harvest point in Bordetella pertussis cultivation for vaccine production. Biotechnology and bioengineering. 2009;103(5):900–8. 10.1002/bit.22326 . 19405154

[13] Nakamura MM, Liew SY, Cummings CA, Brinig MM, Dieterich C, Relman DA. Growth phase- and nutrient limitation-associated transcript abundance regulation in Bordetella pertussis. Infection and immunity. 2006;74(10):5537–48. 10.1128/IAI.00781-06 16988229 PMC1594893

[14] Decker KB, James TD, Stibitz S, Hinton DM. The Bordetella pertussis model of exquisite gene control by the global transcription factor BvgA. Microbiology. 2012;158(Pt 7):1665–76. 10.1099/mic.0.058941-0 22628479 PMC3542142

[15] de Gouw D, Diavatopoulos DA, Bootsma HJ, Hermans PW, Mooi FR. Pertussis: a matter of immune modulation. FEMS microbiology reviews. 2011;35(3):441–74. 10.1111/j.1574-6976.2010.00257.x . 21204863

[16] Martinez de Tejada G, Cotter PA, Heininger U, Camilli A, Akerley BJ, Mekalanos JJ, et al Neither the Bvg- phase nor the vrg6 locus of Bordetella pertussis is required for respiratory infection in mice. Infection and immunity. 1998;66(6):2762–8. 9596745 10.1128/iai.66.6.2762-2768.1998 PMC108267

[17] Hamstra HJ, Kuipers B, Schijf-Evers D, Loggen HG, Poolman JT. The purification and protective capacity of Bordetella pertussis outer membrane proteins. Vaccine. 1995;13(8):747–52. . 7483790 10.1016/0264-410x(94)00040-t

[18] Bellalou J, Relyveld EH. Studies on culture conditions of Bordetella pertussis and relationship to immunogenicity of vaccines. Annales de microbiologie. 1984;135B(1):101–10. . 6095715 10.1016/s0769-2609(84)80047-2

[19] Iwasaki A, Medzhitov R. Toll-like receptor control of the adaptive immune responses. Nature immunology. 2004;5(10):987–95. 10.1038/ni1112 . 15454922

[20] Fedele G, Spensieri F, Palazzo R, Nasso M, Cheung GY, Coote JG, et al Bordetella pertussis commits human dendritic cells to promote a Th1/Th17 response through the activity of adenylate cyclase toxin and MAPK-pathways. PloS one. 2010;5(1):e8734 Epub 2010/01/22. 10.1371/journal.pone.0008734 20090944 PMC2806909

[21] Dunne A, Mielke LA, Allen AC, Sutton CE, Higgs R, Cunningham CC, et al A novel TLR2 agonist from Bordetella pertussis is a potent adjuvant that promotes protective immunity with an acellular pertussis vaccine. Mucosal immunology. 2014 10.1038/mi.2014.93 . 25315966

[22] Fransen F, Stenger RM, Poelen MC, van Dijken HH, Kuipers B, Boog CJ, et al Differential effect of TLR2 and TLR4 on the immune response after immunization with a vaccine against Neisseria meningitidis or Bordetella pertussis. PloS one. 2010;5(12):e15692 10.1371/journal.pone.0015692 21203418 PMC3009743

[23] Higgins SC, Jarnicki AG, Lavelle EC, Mills KH. TLR4 mediates vaccine-induced protective cellular immunity to Bordetella pertussis: role of IL-17-producing T cells. J Immunol. 2006;177(11):7980–9. . 17114471 10.4049/jimmunol.177.11.7980

[24] Marr N, Hajjar AM, Shah NR, Novikov A, Yam CS, Caroff M, et al Substitution of the Bordetella pertussis lipid A phosphate groups with glucosamine is required for robust NF-kappaB activation and release of proinflammatory cytokines in cells expressing human but not murine Toll-like receptor 4-MD-2-CD14. Infection and immunity. 2010;78(5):2060–9. Epub 2010/02/24. 10.1128/IAI.01346-09 20176798 PMC2863497

[25] Geurtsen J, Angevaare E, Janssen M, Hamstra HJ, ten Hove J, de Haan A, et al A novel secondary acyl chain in the lipopolysaccharide of Bordetella pertussis required for efficient infection of human macrophages. The Journal of biological chemistry. 2007;282(52):37875–84. 10.1074/jbc.M706391200 . 17967899

[26] Shah NR, Albitar-Nehme S, Kim E, Marr N, Novikov A, Caroff M, et al Minor modifications to the phosphate groups and the C3' acyl chain length of lipid A in two Bordetella pertussis strains, BP338 and 18–323, independently affect Toll-like receptor 4 protein activation. The Journal of biological chemistry. 2013;288(17):11751–60. 10.1074/jbc.M112.434365 23467413 PMC3636864

[27] Marr N, Tirsoaga A, Blanot D, Fernandez R, Caroff M. Glucosamine found as a substituent of both phosphate groups in Bordetella lipid A backbones: role of a BvgAS-activated ArnT ortholog. Journal of bacteriology. 2008;190(12):4281–90. Epub 2008/04/22. 10.1128/JB.01875-07 18424515 PMC2446747

[28] Hoonakker ME, Verhagen LM, Hendriksen CF, van Els CA, Vandebriel RJ, Sloots A, et al In vitro innate immune cell based models to assess whole cell Bordetella pertussis vaccine quality: a proof of principle. Biologicals: journal of the International Association of Biological Standardization. 2015;43(2):100–9. 10.1016/j.biologicals.2014.12.002 . 25633359

[29] Thalen M, van den IJ, Jiskoot W, Zomer B, Roholl P, de Gooijer C, et al Rational medium design for Bordetella pertussis: basic metabolism. Journal of biotechnology. 1999;75(2–3):147–59. . 10553654 10.1016/s0168-1656(99)00155-8

[30] Thalen M, Venema M, van den IJ, Berwald L, Beuvery C, Martens D, et al Effect of relevant culture parameters on Pertussis Toxin expression by Bordetella pertussis. Biologicals: journal of the International Association of Biological Standardization. 2006;34(3):213–20. 10.1016/j.biologicals.2005.11.002 . 16497513

[31] Ziegler-Heitbrock HW, Thiel E, Futterer A, Herzog V, Wirtz A, Riethmuller G. Establishment of a human cell line (Mono Mac 6) with characteristics of mature monocytes. International journal of cancer Journal international du cancer. 1988;41(3):456–61. Epub 1988/03/15. . 3162233 10.1002/ijc.2910410324

[32] Westphal O, Jann K. Bacterial lipopolysaccharides. Extraction with phenol-water and further applications of the procedure. Methods in Carbohydrate Chemistry. 1965:83–91.

[33] Wilm MS, Mann M. Electrospray and Taylor-Cone theory, Dole's beam of macromolecules at last? Int J Mass Spectrom Ion Proc. 1994;136 (2–3):167–80.

[34] Kondakov A, Lindner B. Structural characterization of complex bacterial glycolipids by Fourier transform mass spectrometry. European journal of mass spectrometry. 2005;11(5):535–46. 10.1255/ejms.721 . 16322660

[35] Caroff M, Brisson J, Martin A, Karibian D. Structure of the Bordetella pertussis 1414 endotoxin. FEBS letters. 2000;477(1–2):8–14. . 10899302 10.1016/s0014-5793(00)01720-8

[36] Baart GJ, Willemsen M, Khatami E, de Haan A, Zomer B, Beuvery EC, et al Modeling Neisseria meningitidis B metabolism at different specific growth rates. Biotechnology and bioengineering. 2008;101(5):1022–35. 10.1002/bit.22016 . 18942773

[37] Higgs R, Higgins SC, Ross PJ, Mills KH. Immunity to the respiratory pathogen Bordetella pertussis. Mucosal immunology. 2012;5(5):485–500. 10.1038/mi.2012.54 . 22718262

[38] Flo TH, Halaas O, Torp S, Ryan L, Lien E, Dybdahl B, et al Differential expression of Toll-like receptor 2 in human cells. Journal of leukocyte biology. 2001;69(3):474–81. . 11261796

[39] LeBouder E, Rey-Nores JE, Raby AC, Affolter M, Vidal K, Thornton CA, et al Modulation of neonatal microbial recognition: TLR-mediated innate immune responses are specifically and differentially modulated by human milk. J Immunol. 2006;176(6):3742–52. . 16517743 10.4049/jimmunol.176.6.3742

[40] Shah NR, Hancock RE, Fernandez RC. Bordetella pertussis Lipid A Glucosamine Modification Confers Resistance to Cationic Antimicrobial Peptides and Increases Resistance to Outer Membrane Perturbation. Antimicrobial agents and chemotherapy. 2014;58(8):4931–4. 10.1128/AAC.02590-14 . 24867963 PMC4136009

[41] Allen AG, Thomas RM, Cadisch JT, Maskell DJ. Molecular and functional analysis of the lipopolysaccharide biosynthesis locus wlb from Bordetella pertussis, Bordetella parapertussis and Bordetella bronchiseptica. Molecular microbiology. 1998;29(1):27–38. . 9701800 10.1046/j.1365-2958.1998.00878.x

[42] Harvill ET, Preston A, Cotter PA, Allen AG, Maskell DJ, Miller JF. Multiple roles for Bordetella lipopolysaccharide molecules during respiratory tract infection. Infection and immunity. 2000;68(12):6720–8. 11083787 10.1128/iai.68.12.6720-6728.2000 PMC97772

[43] Preston A, Thomas R, Maskell DJ. Mutational analysis of the Bordetella pertussis wlb LPS biosynthesis locus. Microbial pathogenesis. 2002;33(3):91–5. . 12220985 10.1006/mpat.2002.0511

[44] Robins LI, Williams AH, Raetz CR. Structural basis for the sugar nucleotide and acyl-chain selectivity of Leptospira interrogans LpxA. Biochemistry. 2009;48(26):6191–201. 10.1021/bi900629e 19456129 PMC2710806

[45] Parkhill J, Sebaihia M, Preston A, Murphy LD, Thomson N, Harris DE, et al Comparative analysis of the genome sequences of Bordetella pertussis, Bordetella parapertussis and Bordetella bronchiseptica. Nature genetics. 2003;35(1):32–40. 10.1038/ng1227 . 12910271

[46] Allen A, Maskell D. The identification, cloning and mutagenesis of a genetic locus required for lipopolysaccharide biosynthesis in Bordetella pertussis. Molecular microbiology. 1996;19(1):37–52. . 8821935 10.1046/j.1365-2958.1996.354877.x

[47] Allen AG, Isobe T, Maskell DJ. Identification and cloning of waaF (rfaF) from Bordetella pertussis and use to generate mutants of Bordetella spp. with deep rough lipopolysaccharide. Journal of bacteriology. 1998;180(1):35–40. 9422589 10.1128/jb.180.1.35-40.1998 PMC106845

[48] White KA, Kaltashov IA, Cotter RJ, Raetz CR. A mono-functional 3-deoxy-D-manno-octulosonic acid (Kdo) transferase and a Kdo kinase in extracts of Haemophilus influenzae. The Journal of biological chemistry. 1997;272(26):16555–63. . 9195966 10.1074/jbc.272.26.16555

[49] White KA, Lin S, Cotter RJ, Raetz CR. A Haemophilus influenzae gene that encodes a membrane bound 3-deoxy-D-manno-octulosonic acid (Kdo) kinase. Possible involvement of kdo phosphorylation in bacterial virulence. The Journal of biological chemistry. 1999;274(44):31391–400. . 10531340 10.1074/jbc.274.44.31391

[50] Geurtsen J, Dzieciatkowska M, Steeghs L, Hamstra HJ, Boleij J, Broen K, et al Identification of a novel lipopolysaccharide core biosynthesis gene cluster in Bordetella pertussis, and influence of core structure and lipid A glucosamine substitution on endotoxic activity. Infection and immunity. 2009;77(7):2602–11. Epub 2009/04/15. 10.1128/IAI.00033-09 19364841 PMC2708539

[51] Merkel TJ, Stibitz S, Keith JM, Leef M, Shahin R. Contribution of regulation by the bvg locus to respiratory infection of mice by Bordetella pertussis. Infection and immunity. 1998;66(9):4367–73. 9712789 10.1128/iai.66.9.4367-4373.1998 PMC108527

[52] Weiss AA, Falkow S. Genetic analysis of phase change in Bordetella pertussis. Infection and immunity. 1984;43(1):263–9. 6317569 10.1128/iai.43.1.263-269.1984 PMC263420

[53] Robinson A, Gorringe AR, Funnell SG, Fernandez M. Serospecific protection of mice against intranasal infection with Bordetella pertussis. Vaccine. 1989;7(4):321–4. . 2573215 10.1016/0264-410x(89)90193-x

[54] de Gouw D, Hermans PW, Bootsma HJ, Zomer A, Heuvelman K, Diavatopoulos DA, et al Differentially Expressed Genes in Bordetella pertussis Strains Belonging to a Lineage Which Recently Spread Globally. PloS one. 2014;9(1):e84523 10.1371/journal.pone.0084523 24416242 PMC3885589

[55] Mills KH, Ryan M, Ryan E, Mahon BP. A murine model in which protection correlates with pertussis vaccine efficacy in children reveals complementary roles for humoral and cell-mediated immunity in protection against Bordetella pertussis. Infection and immunity. 1998;66(2):594–602. 9453614 10.1128/iai.66.2.594-602.1998 PMC107945

[56] Roberts M, Tite JP, Fairweather NF, Dougan G, Charles IG. Recombinant P.69/pertactin: immunogenicity and protection of mice against Bordetella pertussis infection. Vaccine. 1992;10(1):43–8. . 1539459 10.1016/0264-410x(92)90418-j

[57] Fedele G, Nasso M, Spensieri F, Palazzo R, Frasca L, Watanabe M, et al Lipopolysaccharides from Bordetella pertussis and Bordetella parapertussis differently modulate human dendritic cell functions resulting in divergent prevalence of Th17-polarized responses. J Immunol. 2008;181(1):208–16. Epub 2008/06/21. . 18566386 10.4049/jimmunol.181.1.208

[58] Weyrich LS, Feaga HA, Park J, Muse SJ, Safi CY, Rolin OY, et al Resident microbiota affect Bordetella pertussis infectious dose and host specificity. The Journal of infectious diseases. 2014;209(6):913–21. 10.1093/infdis/jit597 24227794 PMC3935476

[59] Trent MS, Ribeiro AA, Lin S, Cotter RJ, Raetz CR. An inner membrane enzyme in Salmonella and Escherichia coli that transfers 4-amino-4-deoxy-L-arabinose to lipid A: induction on polymyxin-resistant mutants and role of a novel lipid-linked donor. The Journal of biological chemistry. 2001;276(46):43122–31. 10.1074/jbc.M106961200 . 11535604

